# Analysis of Polyphenol Extract from Hazel Leaf and Ameliorative Efficacy and Mechanism against Hyperuricemia Zebrafish Model via Network Pharmacology and Molecular Docking

**DOI:** 10.3390/molecules29020317

**Published:** 2024-01-08

**Authors:** Xinhe Wang, Jiarui Zhao, Zhi Lin, Jun Li, Xiaowen Li, Xinyi Xu, Yuchen Wang, Guangfu Lv, He Lin, Zhe Lin

**Affiliations:** College of Pharmacy, Changchun University of Chinese Medicine, Changchun 130117, China; m17704303262_1@163.com (X.W.); zjr0852@163.com (J.Z.); linzhi1@jxutcm.edu.cn (Z.L.); 18224088551@163.com (J.L.); xiaowenli0413@163.com (X.L.); xucynthia@163.com (X.X.); wycscar@hotmail.com (Y.W.); lvgf@ccucm.edu.cn (G.L.)

**Keywords:** hazel leaf, polyphenol extract, hyperuricemia, zebrafish

## Abstract

Hazel leaf, a by-product of hazelnuts, is commonly used in traditional folk medicine in Portugal, Sweden, Iran and other regions for properties such as vascular protection, anti-bleeding, anti-edema, anti-infection, and pain relief. Based on our previous studies, the polyphenol extract from hazel leaf was identified and quantified via HPLC fingerprint. The contents of nine compounds including kaempferol, chlorogenic acid, myricetin, caffeic acid, p-coumaric acid, resveratrol, luteolin, gallic acid and ellagic acid in hazel leaf polyphenol extract (ZP) were preliminary calculated, among which kaempferol was the highest with 221.99 mg/g, followed by chlorogenic acid with 8.23 mg/g. The inhibition of ZP on α-glucosidase and xanthine oxidase activities was determined via the chemical method, and the inhibition on xanthine oxidase was better. Then, the effect of ZP on hyperuricemia zebrafish was investigated. It was found that ZP obviously reduced the levels of uric acid, xanthine oxidase, urea nitrogen and creatinine, and up-regulated the expression ofOAT1 and HPRT genes in hyperuricemia zebrafish. Finally, the targeted network pharmacological analysis and molecular docking of nine polyphenol compounds were performed to search for relevant mechanisms for alleviating hyperuricemia. These results will provide a valuable basis for the development and application of hazel leaf polyphenols as functional ingredients.

## 1. Introduction

Hazelnut belongs to the genus Hazel of the birch family, which is native to Europe and Western Asia and also cultivated in North America [1]. Hazelnuts are one of the most popular nuts in the world, containing nutrients and bioactive ingredients such as protein, fatty acids, dietary fiber and polyphenols [2,3]. Soluble dietary fiber extracted from hazelnut kernel has ideal water retention, oil retention and swelling reduction properties, which can alleviate hyperlipidemia and obesity [4]. Hazelnut oil contains a high content of phytosterols such as oleic acid, tocopherol and tocotrienol, which can induce colon cancer cell death by regulating the bax/bcl-2 transfer mechanism [5]. In recent years, with the increasing cultivation area of hazelnuts, a large number of by-products have been produced, including hazel husks, shells and leaves [2,6]. Due to the preference of modern agriculture and food processing industry for the kernel part of hazelnut, its by-products are considered waste and lack optimal utilization, resulting in more waste and greater resource consumption [7]. Therefore, in order to avoid the pressure caused by a large amount of hazelnut by-products on the environment, it is necessary to comprehensively research these by-products and use them to solve some problems in human life. Hazel leaf, one of the by-products of hazelnut, is widely used in traditional and folk medicine in many countries and regions of the world. In traditional Iranian medicine, hazel leaves are used as a liver tonic and for the treatment of hemorrhoids, varicose veins, phlebitis and mild edema in folk medicine [3]. In traditional Swedish medicine, hazel leaves are used to treat pain. They are also used in Portuguese folk medicine to treat varicose veins and hemorrhoids. Current studies and our previous studies have shown that the main components of hazel leaves are polyphenols, which have obvious antioxidant, anti-inflammatory and antibacterial activities [8]. 

Polyphenols, as the largest group of phytochemicals, are widely present in plant-based foods such as fruits and vegetables. Numerous studies have shown that a diet rich in fruits and vegetables is negatively associated with diseases caused by oxidative stress, such as cardiovascular disease, obesity, diabetes and hyperuricemia [9]. Among them, hyperuricemia (HUA) is a metabolic disease caused by excessive production and abnormal metabolism of uric acid in the body, which may lead to gout, cardiovascular diseases and kidney failure [10]. It has been reported that uric acid can activate the inflammasome pathway through urate crystals produced by intracellular lysosome rupture and reactive oxygen species produced by mitochondria. Additionally, oxidative stress induced by xanthine oxidase can directly or indirectly increase serum uric acid concentration [11]. In this paper, the main components of hazel leaf polyphenol extract were quantitatively analyzed, and the effects of hazel leaf polyphenols on hyperuricemia and the related mechanisms were verified using a hyperuricemia zebrafish model and targeted network pharmacology. This may provide a further experimental basis for the application of hazel leaf polyphenols in functional foods or drug additives. 

## 2. Results

### 2.1. HPLC Analysis

**Figure 1:**
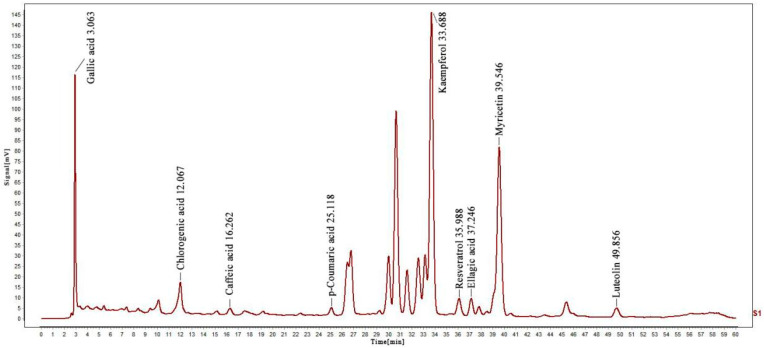
HPLC analysis of nine phenolic compounds in ZP.

The presence and content of phenolic compounds were determined using the HPLC method (Figure S1). Thus, different standard phenolic compounds, namely resveratrol, p-Coumaric acid, caffeic acid, chlorogenic acid, gallic acid, luteolin, ellagic acid, kaempferol, and myricetin, were analyzed within the elution time range of 3.063, 12.067, 16.262, 25.118, 33.688, 35.988, 37.246, 39.546 and 49.856 min, as shown in Figure 1. The content of various determined compounds in ZP was also calculated by peak area, and the results are shown in Table 1. According to the data, the highest content of kaempferol was 221.9947 mg/g, followed by chlorogenic acid and populin, with the lowest content of p-coumaric acid being only 0.0179 mg/g. 

### 2.2. Inhibition of α-GLU

**Figure 2:**
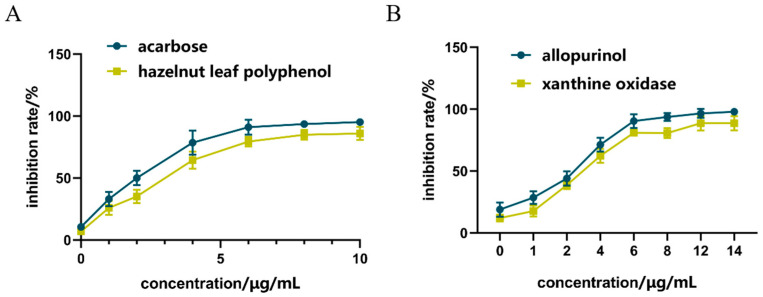
Inhibition of ZP on α-GLU and XOD in vitro, (**A**) α-GLU inhibition; (**B**) XOD inhibition.

As shown in Figure 2A, compared with acarbose in the positive control group, the inhibitory effect of hazel leaf polyphenol extract increased from 0.59% to 82.86% in the concentration range of 0–10 μg/mL, and the inhibitory effect gradually reached the maximum value at 8 μg/mL. The IC50 of hazel leaf polyphenol extract was 0.3886 nM, which was higher than the control value of 0.2158 nM. These results suggested that hazel leaf polyphenol extract has a good inhibitory effect on α-GLU, but not as good as that of acarbose. 

### 2.3. Inhibition of XOD

As shown in Figure 2B, compared with allopurinol in the positive control group, the inhibition of hazel leaf polyphenol extract increased from 11% to 93% in the concentrations range of 0–14 μg/mL, and the inhibition reached the maximum value at 12 μg/mL. The inhibition at the concentration of 14 μg/mL was equivalent to that at 12 μg/mL. The IC50 of hazel leaf polyphenol extract was 8.257 nM, which was lower than the IC50 value of 9.538 nM in the control group. These results indicated that hazel leaf polyphenol extract has a very significant inhibitory effect on XOD, even better than the positive control allopurinol. 

### 2.4. Effects of ZP on Hyperuricemia Zebrafish

#### 2.4.1. Effects of ZP on UA and XOD in Hyperuricemia Zebrafish

**Figure 3:**
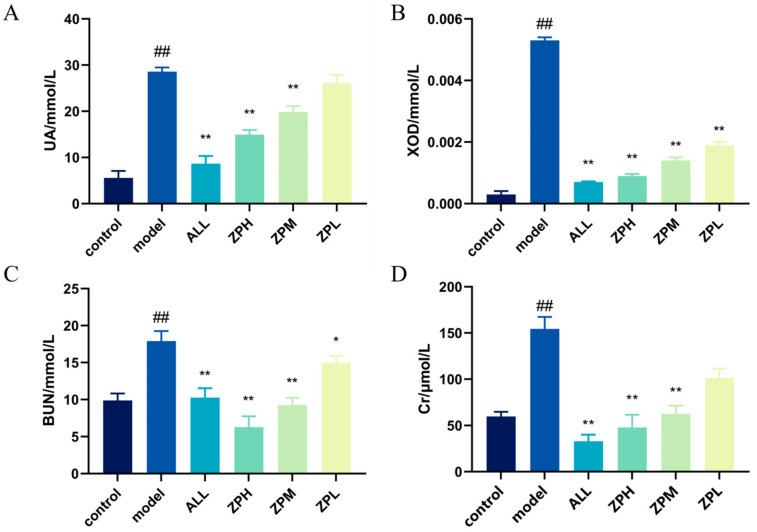
Effects of ZP on UA, XOD, BUN and Cr in hyperuricaemic zebrafish, (**A**) UA level; (**B**) XOD level; (**C**) BUN level; (**D**) Cr level, ^##^ *p* < 0.01 compared to control group. * *p* < 0.05, ** *p* < 0.01 compared to model group.

The level of UA in the body is an important index in the diagnosis of clinical hyperuricemia [12]. As shown in Figure 3A, compared with the control group, the UA level of hyperuricemia zebrafish was significantly increased (*p* < 0.01). Compared with the model group, allopurinol significantly reduced UA levels in hyperuricemia zebrafish (*p* < 0.01). The ZPH and ZPM groups also showed a significant decrease in the UA level (*p* < 0.01). However, there was no significant difference in the ZPL group. The results indicated that hazel leaf polyphenol extract could reduce the content of UA in vivo. 

The XOD can oxidize hypoxanthine and xanthine to UA in vivo [13]. Therefore, we detected the level of XOD in hyperuricemia zebrafish. As shown in Figure 3B, compared with the control group, the XOD level was evidently higher in hyperuricemia zebrafish (*p* < 0.01). Compared with the model group, allopurinol evidently reduced XOD levels in hyperuricemia zebrafish (*p* < 0.01). The ZPH, ZPM and ZPL groups also showed a significant decrease in the XOD level (*p* < 0.01). The results indicated that hazel leaf polyphenol extract had good XOD inhibition, and the effect of the high-dose group was similar to that of allopurinol. 

#### 2.4.2. Effects of ZP on BUN and Cr in Hyperuricemia Zebrafish

BUN and Cr are important indicators of kidney injury, which can determine whether hyperuricemia causes kidney injury [14]. As shown in Figure 3C,D, BUN and Cr levels were significantly higher in the model group compared to the control group (*p* < 0.01), indicating that elevated uric acid caused kidney damage. Compared with the model group, allopurinol significantly reduced BUN and Cr levels in hyperuricemia zebrafish (*p* < 0.01). The levels of BUN and Cr in the ZPH and ZPM groups were also significantly decreased (*p* < 0.01). The level of BUN in the ZPL group decreased significantly (*p* < 0.05), but there was no significant difference in Cr level. The results suggested that hazel leaf polyphenol extract could alleviate the renal injury caused by high uric acid in zebrafish. 

#### 2.4.3. Effects of ZP on the Expression of Genes OAT1 and HPRT1 in Hyperuricemia Zebrafish

**Figure 4:**
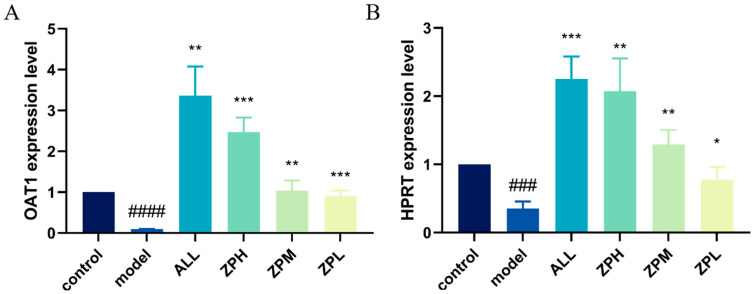
Effects of ZP on the expression of genes OAT1 and HPRT1 in hyperuricemia zebrafish, (**A**) OAT1 gene; (**B**) HPRT gene, ^###^ *p* < 0.001, ^####^ *p* < 0.0001 compared to control group. * *p* < 0.05, ** *p* < 0.01, *** *p* < 0.001 compared to model group.

OAT1 is a drug transporter primarily present in the kidneys with a primary function of excreting uric acid [15]. HPRT1 is the most important enzyme in the purine remediation pathway. Its function defect leads to the breakdown of excess purine into uric acid, which is the main reason for the increase in blood uric acid concentration [16]. As shown in Figure 4A,B, the expression of OAT1 and HPRT genes was significantly decreased in the model group compared to the control group. Compared with the model group, the expression of OAT1 and HPRT1 genes was significantly up-regulated in the ZPH, ZPM and ZPL groups (*p* < 0.01). The results indicated that hazel leaf polyphenol extract could effectively up-regulate the expression of the above two genes. 

### 2.5. Network Pharmacology Analysis

#### 2.5.1. Predicted Component and Disease Targets

**Figure 5:**
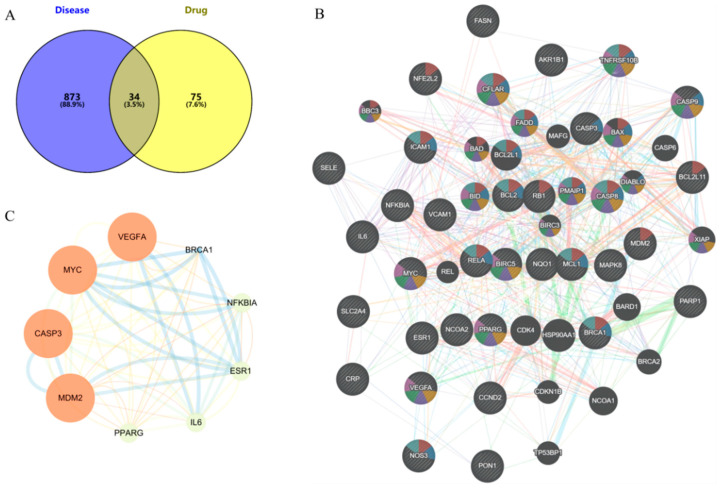
Targets analysis of network pharmacology, (**A**) Common targets, (**B**) PPI of common targets, (**C**) Core targets (larger nodes and deeper color indicate higher degree value).

The TCMSP database was used to screen the potential targets of nine phenolic compounds. A total of 109 drug targets were obtained after removing duplicate values. The HUA targets in GeneCards database were mined, and 907 disease targets were obtained after screening and summary and removing duplicate values. The intersection of drug targets and disease targets was mapped, and 34 intersection targets were obtained (Figure 5A). 

#### 2.5.2. PPI Network

The intersection targets were imported into the STRING database to build the initial PPI network. As shown in Figure 5B, the PPI network had 34 targets, 34 nodes, and 289 edges, with an average node degree of 17, local clustering coefficient of 0.779, and PPI enrichment *p*-value < 1.0 × 10^−16^. The core targets VEGFA, MYC, CASP3, MDM2, PPARG, IL-6, ESR1, NF-ĸB, and BRCA1 were identified by Cytoscape 3.9.0 visualization (Figure 5C). 

#### 2.5.3. GO and KEGG Enrichment Analysis

**Figure 6:**
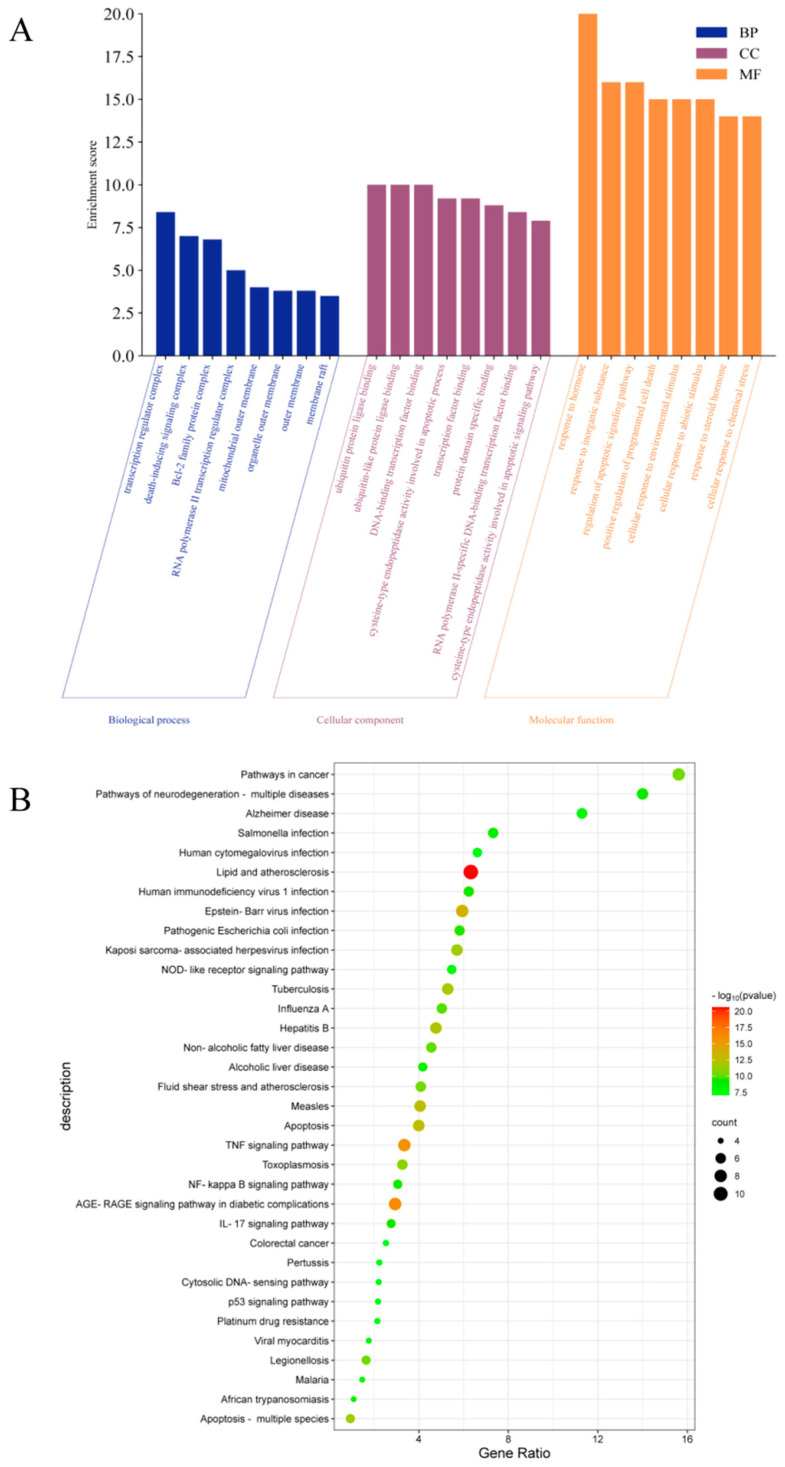
GO and KEGG pathway enrichment analysis, (**A**) BP, CC, and MF according to Go analysis, (**B**) top 35 results of KEGG analysis.

The intersection targets were uploaded to the DAVID database for GO and KEGG pathway enrichment analysis. The analysis data results were sorted according to *p*-value, and *p* < 0.001 was used as the threshold for screening and analysis. GO analysis revealed 498 biological processes (BP), 14 cellular components (CC), and 36 molecular functions (MFs). As shown in Figure 6A, the main BP analysis included the response to hormones, the response to inorganic substances, and the regulation of apoptosis signaling pathways. The main CC analysis included transcriptional regulatory complex, death-inducing signaling complex and Bcl-2 family protein complex. The main MF analysis included ubiquitin protein ligase binding, ubiquitin-like protein ligin binding, and DNA binding transcription factor binding. KEGG analysis enriched the top 35 signaling pathways, including lipid and atherosclerosis, TNF signaling pathway, AGE-RAGE signaling pathway in diabetes complications, NOD-like receptor signaling pathway, apoptosis, NF-κB signaling pathway, IL-17 signaling pathway, p53 signaling pathway and other potential pathways related to HUA (Figure 6B). 

### 2.6. Molecular Docking Analysis

**Figure 7:**
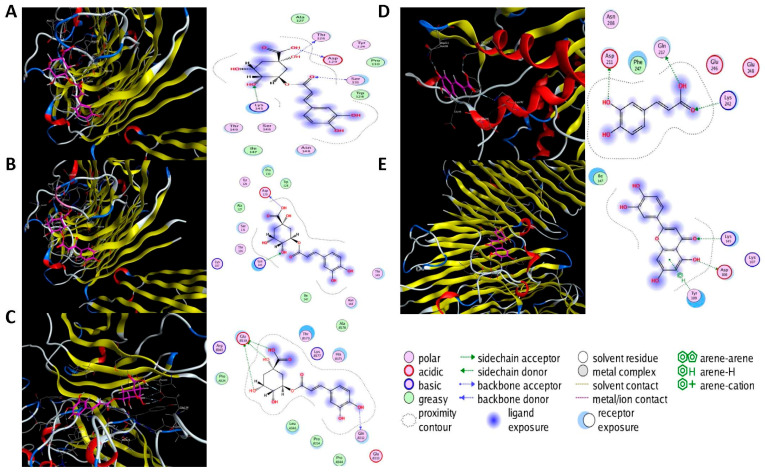
The 3D (left) and 2D (right) binding conformations between the ZP key active ingredient and the core target, (**A**) Chlorogenic acid-BRCA1 (Conformation 1), (**B**) Chlorogenic acid-BRCA1 (Conformation 2), (**C**) Chlorogenic acid-NFKBIA, (**D**) Caffeic acid-CASP3, (**E**) Luteolin-BRCA1.

**Figure 8:**
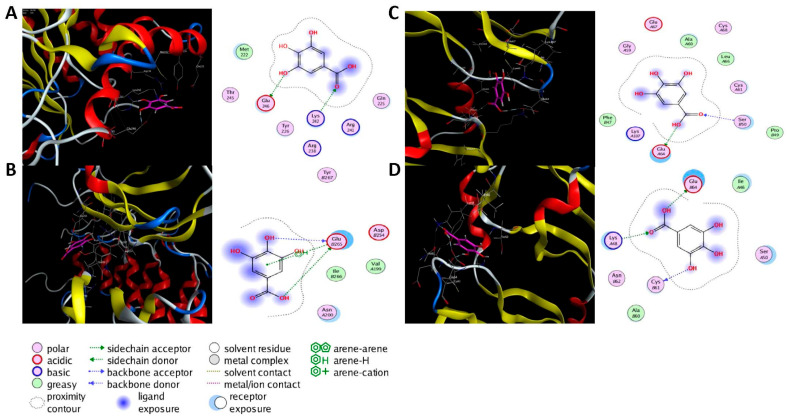
The 3D (left) and 2D (right) binding conformations between the ZP key active ingredient and the core target, (**A**) Gallic acid-CASP3, (**B**) Gallic acid-NFKBIA, (**C**) Gallic acid-VEGFA (Conformation 1), (**D**) Gallic acid-VEGFA (Conformation 2).

Molecular docking was performed on the nine core targets and nine key active components obtained from the previous results. The ligand-receptor binding energy of MOE 2019.0102 is negatively correlated with the affinity. A target-to-target score of less than −7 kcal/mol indicates that the corresponding compound has a high binding ability with the target [17]. Finally, chlorogenic acid, caffeic acid, gallic acid and lutein were screened for further analysis with BRCA1 (PDB ID:1LOB), CASP3 (PDB ID:7RN7), NFKBIA (PDB ID:1IKN) and VEGFA (PDB ID:1MGV). The binding sites and binding forms between compounds and targets are shown in Table 2. As shown in Figure 7A,B, two different conformations of chlorogenic acid bound to BRCA1 through H-acceptor bonds at the lysine C145 binding site, and in Figure 7C, chlorogenic acid bonded to NFKBIA through H-donor at the glutamic acid D138 binding site. Caffeic acid bonded to CASP3 at the lysine B242 binding site in Figure 7D. Luteolin bonded to BRCA1 through H-acceptor bonds at the lysine C145 binding site in Figure 7E. In Figure 8A, gallic acid forms an H-acceptor bond with CASP3 at the lysine binding site. Figure 8B shows that gallic acid formed an H-donor with NFKBIA at the glutamate binding site. Figure 8C,D shows two different conformations of gallic acid bonded to VEGFA through H-donor and H-acceptor bonds at glutamate A64 and lysine A48 binding sites. These results suggested that the four compounds might exert their inhibitory effects on HUA by targeting corresponding targets. 

## 3. Discussion

Hazel leaf is one of the main by-products of hazelnuts. Some studies demonstrated that the extract of hazel leaf contained diarylheptane, flavonol glycosides, kaempferol and yangonin [18]. Another study showed that eight phenolic compounds were identified in the aqueous extracts of three hazel leaves, such as caffeoyl tartaric acid, p-coumaroyl tartaric acid, and populinoid rhamnoside [19]. Similarly, our previous research identified 35 phenolic components including phenolic acids, flavonoids and catechins in the fractions of free, conjugated and bound phenols in hazel leaves [8]. On the basis of previous studies, the content of nine phenolic compounds in hazel leaf polyphenol extract was determined by an external standard method, among which kaempferol was the highest, followed by chlorogenic acid and myricetin. Kaempferol has been shown to protect cerebral ischemia-reperfusion injured cells by inhibiting apoptosis and inhibit iron death by activating the Nrf2/SLC7A11/GPX4 signaling pathway [20]. It was also an estrogen receptor modulator, which could improve atherosclerosis in postmenopausal mice by inhibiting inflammation and apoptosis and activating the G protein-coupled estrogen receptor (GPER) and PI3K/AKT/Nrf2 pathway [21]. It has also shown neuroprotective effects in LPS-induced Parkinson’s disease mice, reducing lipid oxidative stress and mitochondrial damage [22]. Chlorogenic acid alleviated DSS-induced colonic mucosal injury, inflammation and oxidative stress by regulating MAPK/ERK/JNK pathway [23]. Myricetin as a supplement had a positive effect on non-alcoholic fatty liver rats induced by a high-fat diet. It could reduce liver lipid synthesis and inflammation by regulating fecal butyrate-associated intestinal microbiota [24]. However, there have been few studies on the improvement of hyperuricemia by polyphenols in recent years. The inhibition of hazel leaf polyphenol extract on XOD and α-GLU has been confirmed in the earlier stage of this study, and the inhibitory effect of XOD is better than that of α-GLU. XOD is a low-specificity enzyme that can catalyze the production of UA from hypoxanthine and xanthine [25]. Excessive production of UA or blocked excretion of UA in the body may lead to hyperuricemia. Excessive accumulation of urate crystals in the body can lead to gout. Therefore, we further investigated the effect of hazel leaf polyphenol extract on hyperuricemia zebrafish. After hazel leaf polyphenol extract treatment, the levels of UA, XOD, Cr and BUN of hyperuricemia zebrafish were significantly reduced in a dose-dependent manner. HPRT (hypoxanthine-guanine phosphoribosyltransferase) has feedback inhibition on purine synthesis. Its deficiency leads to Lesch Nyhan disease, which is characterized by hyperuricemia, severe movement disorders, chorea, globulin disease, and cognitive and attention deficits. Patients clinically lacking HPRT have varying degrees of gout and neurological disorders [26]. OAT1 (organic anion transporter 1) is a multispecific drug transporter in the kidneys involved in UA excretion [15]. In zebrafish, the similarities of HPRT and OAT1 genes with humans were 91% and 46%, respectively [27]. In this study, HPRT and OAT1 genes were evidently down-regulated in hyperuricemia zebrafish. The expression of the two genes was evidently up-regulated after the administration of hazel leaf polyphenol extract. These results indicated that hazel leaf polyphenol extract could reduce UA production by inhibiting purine synthesis and xanthine oxidase activity, and accelerate its excretion by promoting UA transporters. 

Furthermore, targeted network pharmacology and analytical docking methods were used to explore the molecular mechanism of ZP on HUA. The KEGG analysis suggested that ZP may ameliorate HUA through the TNF signaling pathway, NF-κB signaling pathway, AGE-RAGE signaling pathway and IL-17 signaling pathway. The important feature of HUA was the elevation of UA in the body. Once UA was transferred into cells, it acted as a pro-oxidant, increasing the production of reactive oxygen species (ROS). Sustained oxidative stress could induce the production of inflammatory cytokines, thus affecting multiple organs including kidney injury [28]. TNF-α was mainly produced in peripheral tissues and induced tissue-specific inflammation by participating in ROS production and activation of various transcription-mediated pathways [29]. In renal tubular epithelial cells (NRK-52E) and hyperuricemia mice, UA induced the infiltration of inflammatory cells in the tubulointerstitium, up-regulated the production of inflammatory cytokine TNF-α, and regulated the expression of activated normal T cells and secreted factors through the NF-κB signaling pathway [30]. Other studies have shown that inhibition of AGE-RAGE and IL-17 pathway activation can improve renal inflammation and apoptosis induced by HUA [31]. In addition, we also predicted the core target of HUA by ZP, such as VEGFA, CASP3, PPARG, IL-6, ESR1 and NF-ĸB. It has been shown that neuronal apoptosis and microglia activation in hyperuricemia-mediated cognitive deficits mice are ameliorated by blocking the Myd88/NF-ĸB pathway to modulate the expression of the inflammatory factors IL-6 and the caspase family in mice serum [32]. Caspase-3 was the characteristic target of apoptosis, and down-regulation of caspase-3 could prevent oxidative stress and mitochondrial apoptosis in hyperuricemia-induced nephritic injury [33]. Studies have shown that targeting VEGFA can regulate the STAT3 signaling pathway to inhibit inflammation and oxidative stress, thereby delaying the progression of hyperuricemia-induced chronic dilatation renal fibrosis, improving renal failure, and reducing serum UA levels [34]. PPARG is a member of the nuclear receptor superfamily, which inhibits inflammation mainly through the interaction of the NF-ĸB signaling pathway [35,36]. The genes for estrogen receptors 1 and 2 (ESR1 and ESR2) encode estrogen receptor alpha and estrogen receptor β, respectively [37]. A clinical trial proved the relationship between ESR1 and HUA, and rs712221 polymorphism of the ESR1 gene affected the reduction in serum uric acid level in patients undergoing bariatric surgery [38]. To further investigate the potential molecular mechanism of ZP, we screened four active ingredients and four core targets for molecular docking. It indicated that these ingredients bind BRCA1, CASP3, NFKBIA and VEGFA with high affinity. Chlorogenic acid, caffeic acid, gallic acid and luteolin may be the key compounds for the protective effect of ZP on HUA. These results provided more enlightenment and direction for future research on the treatment of hyperuricemia with hazel leaf polyphenol extract. 

## 4. Materials and Methods

### 4.1. Chemicals and Reagents

Standards of kaempferol, myricetin, chlorogenic acid, caffeic acid, p-coumaric acid, resveratrol, luteolin, gallic acid, and ellagic acid were purchased from Shanghai Yuanye Bio-Technology Co., Ltd. (Shanghai, China). The purity of all standards was required to be greater than or equal to 98%. Potassium oxonate, xanthine oxidase and 2,6-dihydroxypurine were purchased from Shanghai Yuanye Bio-Technology Co., Ltd. (Shanghai, China). Xanthine sodium salt (XSS) and allopurinol (APL) were purchased from Sigma Aldrich Inc. (St. Louis, MO, USA). A uric acid (UA) test kit, xanthine oxidase (XOD) assay kit (Colorimetric method), urea assay kit (BUN), and creatinine (Cr) assay kit (sarcosine oxidase) were purchased from Nanjing Jiancheng Bioengineering Institute (Nanjing, China). 

### 4.2. Sample Preparation

The hazel leaves were freeze-dried and crushed 1.0 g hazel leaf powder was added to 15 mL of a mixed solution of 1% hydrochloric acid and 70% methanol (1:1 volume ratio), vortexed and stirred for 30 s, and extracted using ultrasound at 150 W power for 30 min. We added 2.5 mg cellulase to the obtained solution, adjusted the pH to 3.0, and extracted it in a constant temperature water bath at 50 °C for 80 min. Then, the mixture was centrifuged at 1800× *g* for 10 min and the extraction was repeated twice. The supernatant was combined to obtain hazel leaf polyphenols extract [8]. 

### 4.3. HPLC Fingerprint Analysis

Based on our previous results (Table S1), the samples were analyzed in chromatographic fingerprint by the Agilent 1260II HPLC-UV system equipped with a four-element pump (Santa Clara, CA, USA) with Shimadzu VP-ODS C18 column (150 × 4.6 mm, 5 μm, Kyoto, Japan). The column temperature was maintained at 30 °C. The analyte was eluted at a rate of 1 mL/min using a mobile phase consisting of 0.1% phosphoric acid aqueous solution and methanol under the following conditions as shown in Table 3. 

### 4.4. Antioxidant Assays

#### 4.4.1. α-Glucosidase Assay

In total, 40 μL α-glucosidase (α-GLU) solution and an equal volume sample were added to the reaction system of 96 microtitration plates. The plates were placed in a constant temperature water bath at 37 °C for 10 min and 40 μL 4-Nitrophenyl β-D-glucopyranoside (pNPG) solution was added. Then, the plates were incubated at constant temperature for 30 min. Finally, the reaction was terminated by adding 120 μL sodium carbonate with a concentration of 0.2 mol/L. The absorbance was measured at 405 nm andα-GLU inhibition rate was calculated, with acarbose as a positive control. The IC50 values were calculated by fitting with Graph Pad Prism 7.0 software. Enzyme activity inhibition rate/% = $\frac{A0-(A1-A2)}{A0}\times 100$, A0: the absorbance value of the enzyme after reacting with the substrate; A1: the absorbance value of the enzyme reacting with the substrate after adding the sample; A2: the absorbance value of the sample itself. 

#### 4.4.2. Xanthine Oxidase Assay

In total, 50 μL 0.2 U/mL xanthine oxidase was mixed with an equal volume sample solution and placed at 37 °C for 5 min. The mixture was then added with 150 μL 0.3 mmol/L xanthine solutions (configured with 0.07 mol/L phosphate buffer at pH 7.5) and incubated at 37 °C for 60 min. Finally, the reaction was terminated by adding 80 μL of hydrochloric acid at a concentration of 1 mol/L. The absorbance was measured at 292 nm and the XOD inhibition rate was calculated, with allopurinol as a positive control. The IC50 values were calculated by fitting with Graph Pad Prism 7.0 software. Enzyme activity inhibition rate/% = $\frac{1-(C-D)}{A-B}\times 100$, A: blank test group (enzyme + buffer + substrate); B: blank control group (buffer + substrate); C: sample test group (sample + enzyme + substrate); D: sample control group (sample + buffer + substrate). 

### 4.5. Effects of ZP on Hyperlipemia Zebrafish

#### 4.5.1. Zebrafish Strains and Maintenance

AB wild-type zebrafish were obtained from Nanjing Yishulihua Biotechnology Co., Ltd. (Nanjing, China). All zebrafish embryos were generated from adult pairs maintained at 28 °C in a circulating water system with a 14 h light and a 10 h dark light–dark cycle. Water supplied to the system was filtered by reverse osmosis (pH 7.5–8). Embryos were cultured in a humidity incubator at 28.5 °C. Dead embryos were removed daily. 

#### 4.5.2. Construction and Validation of Hyperuricemia Zebrafish

As the previous method with slight modification [10], 720 juvenile zebrafish were divided into 6 groups, including a control group, model group, positive control group (allopurinol, 13.6 μg/L), hazel leaf polyphenol high-dose group (ZPH, 100 μg/mL), hazel leaf polyphenol medium-dose group (ZPM, 30 μg/mL), and hazel leaf polyphenol low-dose group (ZPL, 10 μg/mL) (Table S2). Three Petri dishes were set for each group with 40 fish in each dish. 

Potassium oxonate 20.00 mM and xanthine sodium salt 1.00 mM were used to establish the zebrafish model of acute hyperuricemia. The control group was exposed to only 20 mL of fish water. The positive group and hazel leaf polyphenol extract groups were given the corresponding dose of the subject to zebrafish larvae, respectively. The zebrafish in each group were kept in a thermostat at 28.5 °C for one day. 

#### 4.5.3. Determination of UA, Cr, BUN and XOD Levels in the Zebrafish

UA, Cr, BUN and XOD in the homogenates of zebrafish larvae were determined by biochemical kits. After one day of simultaneous administration in each group, 100 mg/L of tricaine solution was added to each Petri dish. Approximately 10 min after anesthesia, 40 zebrafish larvae from the same Petri dish were transferred into EP tubes, which required inhaling as much water as possible from the EP tube containing the larvae. Then, 50 mL of ice PBS was added to the EP tubes. All the above procedures were performed on ice. The zebrafish larvae were then homogenized until no visible pieces of tissue remained. The homogenate sample was centrifuged at 15,000 rpm for 10 min at 4 °C. The supernatant was collected for UA, Cr, BUN and XOD determination. 

#### 4.5.4. q-PCR Detection

Total RNA from zebrafish larvae was extracted according to the instructions of the RNAsimple Total RNA Extraction Kit (DP419, TIANGEN, Beijing, China). The qPCR cycles were performed using FastKing One Step Reverse Transcription Fluorescence Quantification Kit (SYBR Green) (FP313, TIANGEN, Beijing, China) according to the suggested reaction steps in the kit instructions. Primer information is shown in Table 4. 

### 4.6. Statistical Analysis

All data were analyzed using SPSS version 24.0 and expressed as mean ± standard deviation. One-way analysis of variance (ANOVA) was performed using *t*-test and ANOVA. The *p*-values of 0.05 and 0.01 were used to indicate statistical significance. 

### 4.7. Network Pharmacology Analysis

#### 4.7.1. Network Target Collection

The TCMSP database was searched for nine of the hazel leaf polyphenol components to identify constituents (https://old.tcmsp-e.com/tcmsp.php, accessed on 5 September 2023). The HUA targets were obtained from GeneCards (https://www.genecards.org/, accessed on 5 September 2023), OMIM database (https://www.omim.org/, accessed on 6 September 2023), DrugBank (https://go.drugbank.com/, accessed on 6 September 2023) and TTD (https://db.idblab.net/TTD/, accessed on 7 September 2023). Finally, Venny 2.1 (https://bioinfogp.cnb.csic.es/tools/venny/index.html, accessed on 8 September 2023) was used to identify overlap targets and HUA targets. 

#### 4.7.2. Protein–Protein Interaction (PPI) Analysis

The PPI network was established using the STRING database version 11.5 (https://cn.string-db.org/, accessed on 8 September 2023). “Homo sapiens” was selected as the organism. Cytoscape 3.9.1 was used to calculate degree centrality (DC), betweenness centrality (BC) and closeness centrality (CC). 

#### 4.7.3. Gene Ontology (GO) and Kyoto Encyclopedia of Genes and Genomes (KEGG) Pathway Enrichment Analysis of Targets

The enrichment of GO terms in the biological process, cellular component and molecular function categories, as well as of KEGG pathway terms, was performed in Metascape (http://metascape.org, accessed on 9 September 2023). When *p* < 0.01, a minimum count of 3 and enrichment factor > 1.5 were considered significant. The top 35 enriched terms were visualized using an online heatmap tool (http://www.bioinformatics.com.cn, accessed on 10 September 2023). 

### 4.8. Molecular Docking Method

Molecular docking was carried out to evaluate the binding affinity between the compounds and their protein target. The structures of protein targets were obtained from the RCSB protein database (PDB) (http://www.rcsb.org/, accessed on 13 September 2023), and small-molecule compounds were obtained from the PubChem database. The target protein was used as the grid center, while the center coordinates (center x/y/z) and box sizes (size x/y/z) were adjusted to cover the protein completely. Molecular docking simulations were performed using Moe 2019.0102. 

## 5. Conclusions

In this study, nine compounds in hazel leaf polyphenol extract were quantified by HPLC based on our previous results. The inhibition of α-GLU and XOD by hazel leaf polyphenol extract was compared in vitro, and the inhibition of XOD was better. It was speculated that hazel leaf polyphenol extract may have a therapeutic effect on HUA. Therefore, a hyperuricemia zebrafish model was constructed, confirming that hazel leaf polyphenol extract effectively reduced the levels of UA, XOD, BUN and Cr, and up-regulated the expression ofOAT1 and HPRT genes in hyperuricemia zebrafish. Finally, the potential molecular mechanism of hazel leaf polyphenol extract was predicted through targeted network pharmacology and molecular docking, providing a foundation for further research on hazel leaf polyphenol extract in the treatment of HUA. It also contributes to the sustainable development and utilization of hazel leaf as a by-product of hazelnut. 

## Figures and Tables

**Table 1:** Content of nine phenolic compounds in ZP.

Compounds	Retention Time/min	Peak Area of Sample	Peak Area of Standard	Concentration of Standard/μg/mL	Quantity Contained/mg/g
Kaempferol	33.688	174,535	13,444	380	221.995
Chlorogenic acid	12.067	23,233	25,414	200	8.228
Myricetin	39.546	131,239	89,580	60	3.956
Ellagic acid	37.246	10,252	32,741	40	0.564
Luteolin	49.856	7840	68,931	50	0.256
Resveratrol	35.988	1998	83,851	200	0.214
Caffeic acid	16.262	4607	37,340	20	0.111
Gallic acid	3.063	52,577	922,439	40	0.103
p-Coumaric acid	25.118	5200	466,775	35.8	0.018

**Table 2:** The binding energy of compounds and core targets (kcal/mol).

Target	PDB ID	Target Structure	Compound	Interaction	Affinity (kcal/mol)	Receptor	Distance
BRCA1	1LOB	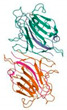	Chlorogenic acid	H-acceptor	−7.6	LYS C145	2.98
					−7.5	LYS C145	2.99
			Luteolin	H-acceptor	−8.3	LYS G145	3.10
CASP3	7RN7	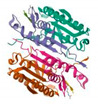	Caffeic acid	H-acceptor	−7.5	LYS B242	2.93
			Gallic acid	H-acceptor	−9.0	LYS B242	3.18
NFKBIA	1IKN	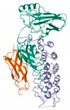	Chlorogenic acid	H-donor	−7.4	GLU D138	−7.4
			Gallic acid	H-donor	−9.4	GLU C265	2.80
VEGFA	1MJV	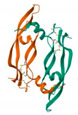	Gallic acid	H-donor	−7.3	GLU A64	2.80
				H-acceptor	−10.2	LYS A48	2.94

**Table 3:** Gradient elution conditions.

Time (min)	0.1% Phosphoric Acid Aqueous Solution (A)	Methanol (B)	Flow Rate (mL/min)
0	80%	20%	1.0
8	75%	25%	1.0
15	70%	30%	1.0
30	60%	40%	1.0
50	55%	45%	1.0
55	40%	60%	1.0
60	80%	20%	1.0

**Table 4:** q-PCR primer information.

Gene Name	Sequence (5′-3′)	Size (bp)
β-actin	TCG AGC AGG AGA TGG GAA CC	26
β-actin	CTC GTG GAT ACC GCA AGA TTC	27
OAT1	GGA CCT GTA AGG CCA GAT CC	26
OAT1	TTG CAG TAG CTT GTC GGT GT	26
HPRT	TTG CAG TAG CTT GTC GGT GT	26
HPRT	CAG ACG TTC AGT TCG GTC CA	26

## Data Availability

Data are contained within the article and Supplementary Materials.

## Supplementary Materials

The following supporting information can be downloaded at: https://www.mdpi.com/article/10.3390/molecules29020317/s1, Table S1. Identification and relative contents of components in hazel leaf polyphenols. Figure S1. Composition of hazel leaf polyphenols versus standards. Table S2. Acute toxicity test of hazel leaf polyphenols in zebrafish.
