# Supplementary material for: Analysis of Polyphenol Extract from Hazel Leaf and Ameliorative Efficacy and Mechanism against Hyperuricemia Zebrafish Model via Network Pharmacology and Molecular Docking
Source: Molecules. 2024 Jan 8;29(2):317. doi: 10.3390/molecules29020317 (PMC10820926; doi:10.3390/molecules29020317)
Supplement: Supplementary file 1 [file molecules-29-00317-s001.zip › molecules-2781628-supplementary.pdf]

# Supplementary Material

**Table S1. Identification and relative contents of components in hazel leaf polyphenols.**

| Compound | Chemical formula | Relative content (×$10^4$) |
|---|---|---|
| Gallic acid | $C_7H_6O_5$ | 31.3±6.22 |
| Gallocatechin | $C_{15}H_{14}O_7$ | 2.79±0.57 |
| 2,5-dihydroxybenzoic acid | $C_7H_6O_4$ | 5.78±1.26 |
| Chlorogenic acid | $C_{16}H_{18}O_9$ | 4.13 ± 0.21 |
| Quercetin-3-O-beta-D-glucopyranoside | $C_{21}H_{20}O_{12}$ | 36.75±7.61 |
| P-Coumaric acid | $C_9H_8O_3$ | 16.33±3.89 |
| Myricetin | $C_{15}H_{10}O_8$ | 74.32±12.87 |
| Pedalitin | $C_{16}H_{12}O_7$ | 4.23 ± 0.91 |
| Quercetin | $C_{15}H_{10}O_7$ | 154.91±24.28 |
| Luteolin-7-O-glucoside | $C_{21}H_{20}O_{11}$ | 73.07±18.04 |
| Caffeic acid | $C_9H_8O_4$ | 14.27±2.07 |
| Resveratrol | $C_{14}H_{12}O_3$ | 4.59 ± 0.52 |
| Luteolin | $C_{15}H_{10}O_6$ | 21.29±6.22 |
| Ellagic acid | $C_{14}H_6O_8$ | 15.79 ± 4.37 |
| Hydroxybenzoic acid | $C_7H_6O_3$ | 6.25±1.30 |
| Quercetin-3-O-beta-D-glucuronide | $C_{21}H_{18}O_{13}$ | 6.43 ± 0.60 |
| Kaempferol-3-O-rhamnoside | $C_{21}H_{20}O_{10}$ | 27.77± 5.36 |

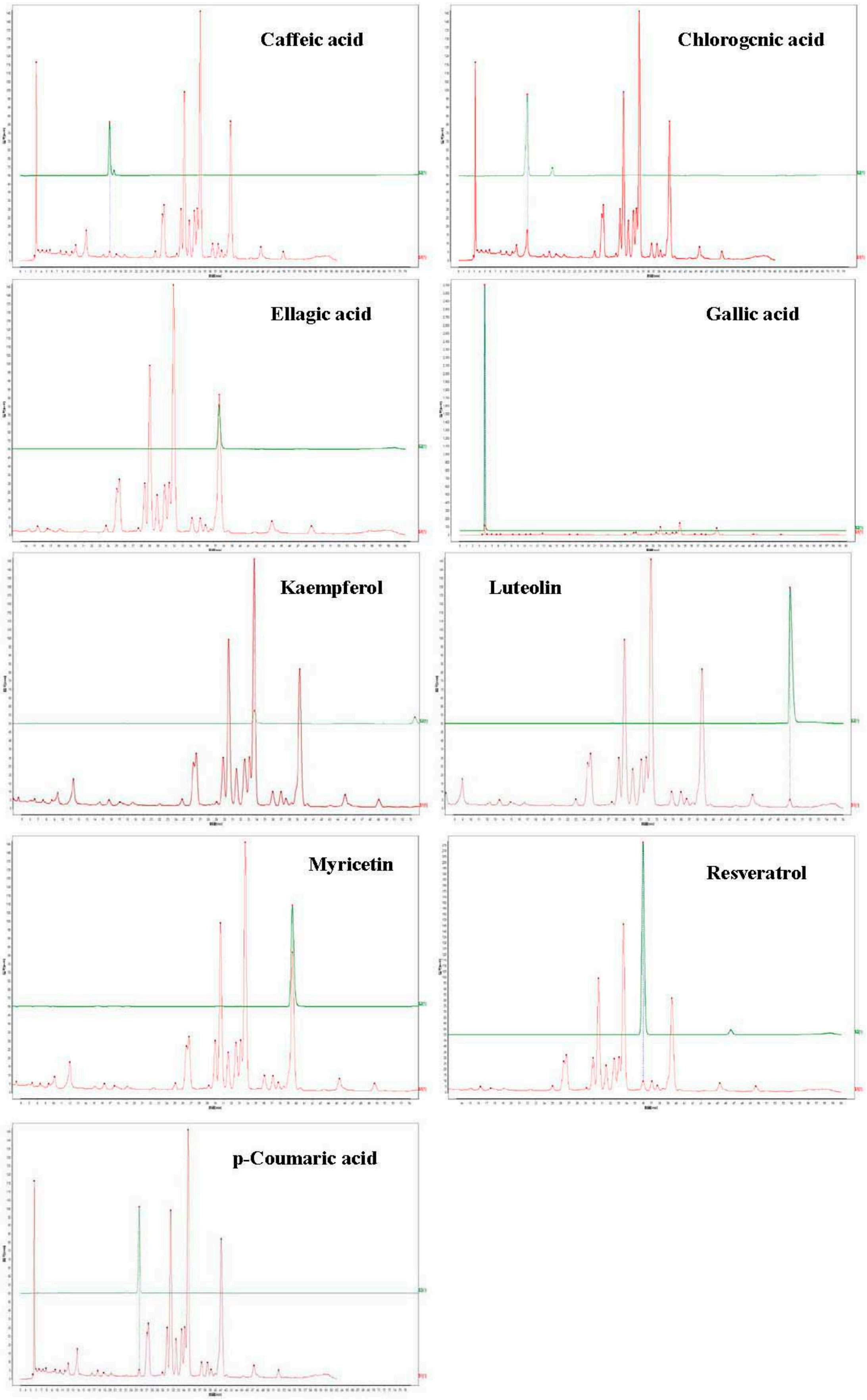


**Figure S1. Composition of hazel leaf polyphenols versus standards.**

**Table S2. Acute toxicity test of hazel leaf polyphenols in zebrafish.**

| Groups | Hazel leaf polyphenols (μg/mL) | Mortality rate (%) |
|---|---|---|
| 1 | 1000 | 98 |
| 2 | 700 | 51 |
| 3 | 500 | 23 |
| 4 | 300 | 16 |
| 5 | 100 | 0 |
